# Biological Characterization of 3-(2-amino-ethyl)-5-[3-(4-butoxyl-phenyl)-propylidene]-thiazolidine-2,4-dione (K145) as a Selective Sphingosine Kinase-2 Inhibitor and Anticancer Agent

**DOI:** 10.1371/journal.pone.0056471

**Published:** 2013-02-20

**Authors:** Kai Liu, Tai L. Guo, Nitai C. Hait, Jeremy Allegood, Hardik I. Parikh, Wenfang Xu, Glen E. Kellogg, Steven Grant, Sarah Spiegel, Shijun Zhang

**Affiliations:** 1 Department of Medicinal Chemistry, Virginia Commonwealth University, Richmond, Virginia, United States of America; 2 Departments of Pharmacology & Toxicology, Virginia Commonwealth University, Richmond, Virginia, United States of America; 3 Department of Biochemistry and Molecular Biology, Virginia Commonwealth University, Richmond, Virginia, United States of America; 4 Department of Internal Medicine and Massey Cancer Center, Virginia Commonwealth University, Richmond, Virginia, United States of America; 5 School of Pharmaceutical Sciences, Shandong University, Shandong, People’s Republic of China; Vanderbilt University, United States of America

## Abstract

In our effort to develop selective sphingosine kinase-2 (SphK2) inhibitors as pharmacological tools, a thiazolidine-2,4-dione analogue, 3-(2-amino-ethyl)-5-[3-(4-butoxyl-phenyl)-propylidene]-thiazolidine-2,4-dione (K145), was synthesized and biologically characterized. Biochemical assay results indicate that K145 is a selective SphK2 inhibitor. Molecular modeling studies also support this notion. *In vitro* studies using human leukemia U937 cells demonstrated that K145 accumulates in U937 cells, suppresses the S1P level, and inhibits SphK2. K145 also exhibited inhibitory effects on the growth of U937 cells as well as apoptotic effects in U937 cells, and that these effects may be through the inhibition of down-stream ERK and Akt signaling pathways. K145 also significantly inhibited the growth of U937 tumors in nude mice by both intraperitoneal and oral administration, thus demonstrating its *in vivo* efficacy as a potential lead anticancer agent. The antitumor activity of K145 was also confirmed in a syngeneic mouse model by implanting murine breast cancer JC cells in BALB/c mice. Collectively, these results strongly encourage further optimization of K145 as a novel lead compound for development of more potent and selective SphK2 inhibitors.

## Introduction

Sphingosine-1-phosphate (S1P), a lipid metabolite, has been recently demonstrated to be an important signaling mediator for vital cellular and physiological processes, such as cell motility, invasion, proliferation, angiogenesis and apoptosis [1], [2]. S1P is produced from sphingosine via phosphorylation by two isoenzymes, sphingosine kinase-1 (SphK1) [3] and sphingosine kinase-2 (SphK2) [4]. Upon production, S1P interacts with a family of G protein-coupled receptors (S1PR_1–5_) on the cell surface [5] and/or intracellular targets, such as histone deacetylase (HDAC) [6] and TRAF2 [7], to play a plethora of roles in diverse pathophysiological conditions such as inflammation, immunity and cancer.

**Figure 1:**
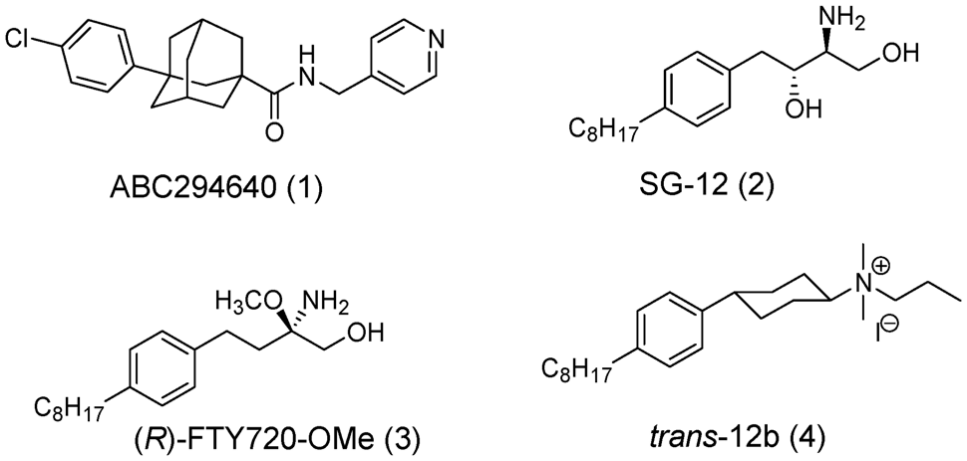
Chemical structures of SphK2 inhibitors.

Ceramide and sphingosine, the precursors of S1P, have been associated with growth arrest and apoptosis [8]. In contrast, S1P has been demonstrated to play pro-survival roles [9]. The regulation of the levels of these metabolites, a so called “sphingolipid rheostat” [1], [9], is complex and a number of enzymes have been demonstrated to be important [2], [8], among which SphK1 and SphK2 have emerged as central players [2], [10]. Although SphK1 and SphK2 share a high degree of homology, they differ significantly in size, tissue distribution, and subcellular localization [11]. For example, SphK1 mainly resides in the cytosol [12] while SphK2 is present in several intracellular compartments, mainly in the nucleus, endoplasmic reticulum, and mitochondria [13]. Evidence has accumulated that SphK1 promotes cell growth and survival, and has been associated with many aspects of cancer development and progression, such as proliferation, migration, invasion and angiogenesis [14]. Consistent with this, numerous studies have shown that SphK1 is frequently up-regulated and/or overexpressed in tumor tissues compared to normal tissues [15]. Much less is known about SphK2. Initially, SphK2 had been demonstrated to be pro-apoptotic as overexpression of SphK2 suppresses growth and promotes apoptosis [16]. However, it was subsequently shown that downregulation of SphK2 inhibits the proliferation and migration of tumor cells, such as glioblastoma and breast cancer cells [17], [18] and ablation of SphK2 or employing a SphK2 inhibitor has been shown to inhibit the xenograft growth of tumor cells in mice [15], [19], [20]. Recently, HDAC has been identified as an intracellular target of S1P, which is mainly produced by SphK2 within the nucleus and indicates a potential role of SphK2 in histone acetylation, gene expression, and cancer pathology [6]. SphK2 has also been demonstrated to play important roles in the function of mitochondria [21]. Even with these very recent advances in understanding of SphK2, much is still unknown or controversial about this kinase. Therefore, development of selective SphK2 inhibitors would be of great value as pharmacological tools to complement the ongoing molecular and genetic studies, and help unravel the roles of SphK2 in different pathological and physiological conditions. Although a number of potent and selective SphK1 inhibitors have been developed and reported [2], [22]–[24], only a few SphK2 inhibitors with moderate potency, such as ABC 294640 (**1**) [19], SG-12 (**2**) [25], R-FTY720-OMe (**3**) [26] and trans-12 (**4**) [27], have been reported (Figure 1). Therefore, it would be of great value to have new and adaptable chemical scaffolds available as selective SphK2 inhibitors as this would help unravel the structural requirements for designing new SphK2 inhibitors.

**Figure 2:**
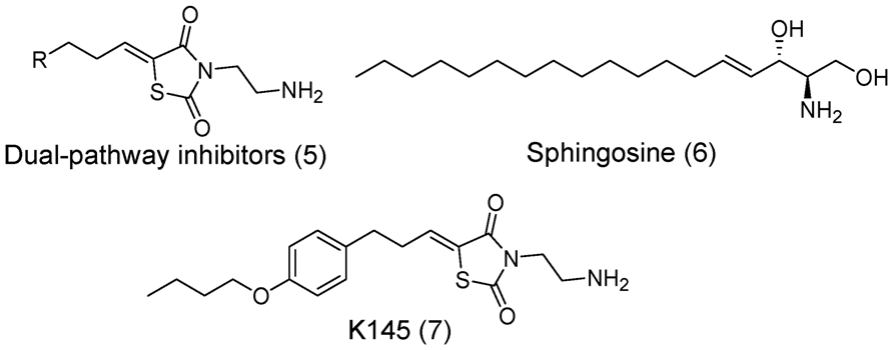
Chemical structures of dual-pathway inhibitors, sphingosine, and K145.

Recently, our research group has initiated development of 3-(2-amino-ethyl)-thiazolidine-2,4-dione (TZD) analogues (**5**, Figure 2) as dual-pathway inhibitors of the ERK and Akt signaling pathways [28], [29]. However, the cellular target(s) of these dual-pathway inhibitors remain unknown. Although the rhodanine- and TZD-compound types have been referred to as Pan Assay INterference compounds (PAINs) because of their frequent appearance as hits suggesting promiscuity [30], rhodanine and TZD analogues have also been recognized as privileged templates in drug design and discovery [31]. Recently, studies have also suggested that distinct, not nonsepecific, interactions exist between them and biomacromolecules, and that these scaffolds should not be regarded as promiscuous binders, although diligence in examining selectivity for moderate affinity compounds with these functional groups is advised [32]. Numerous compounds containing the TZD ring have already been developed as potential anticancer agents, such as the PI3K inhibitor GSK1059615 and its analogues [33].

In comparing it to sphingosine (**6**), the 3-(2-amino-ethyl)-TZD moiety of our dual-pathway inhibitors may be able to mimic the amino-hydroxyl sphingoid base. Furthermore, SphK inhibitors have been shown to inhibit the ERK and Akt signaling pathways as a downstream event of SphK inhibition [10], [23], [24]. These observations suggested to us that SphKs might be protein targets of the TZD-based dual-pathway inhibitors. Furthermore, previous studies suggested that a phenyl ring with an alkyl chain is an important structural feature as SphK inhibitors [24]. Therefore, we report herein the design and biological characterization of **K145**, 3-(amino-ethyl)-5-[3-(4-butoxyl-phenyl)-propylidene]-thiazolidine-2,4-dione (**7**, Figure 2) as a selective SphK2 inhibitor.

## Results and Discussion

### Chemistry

**Figure 3:**
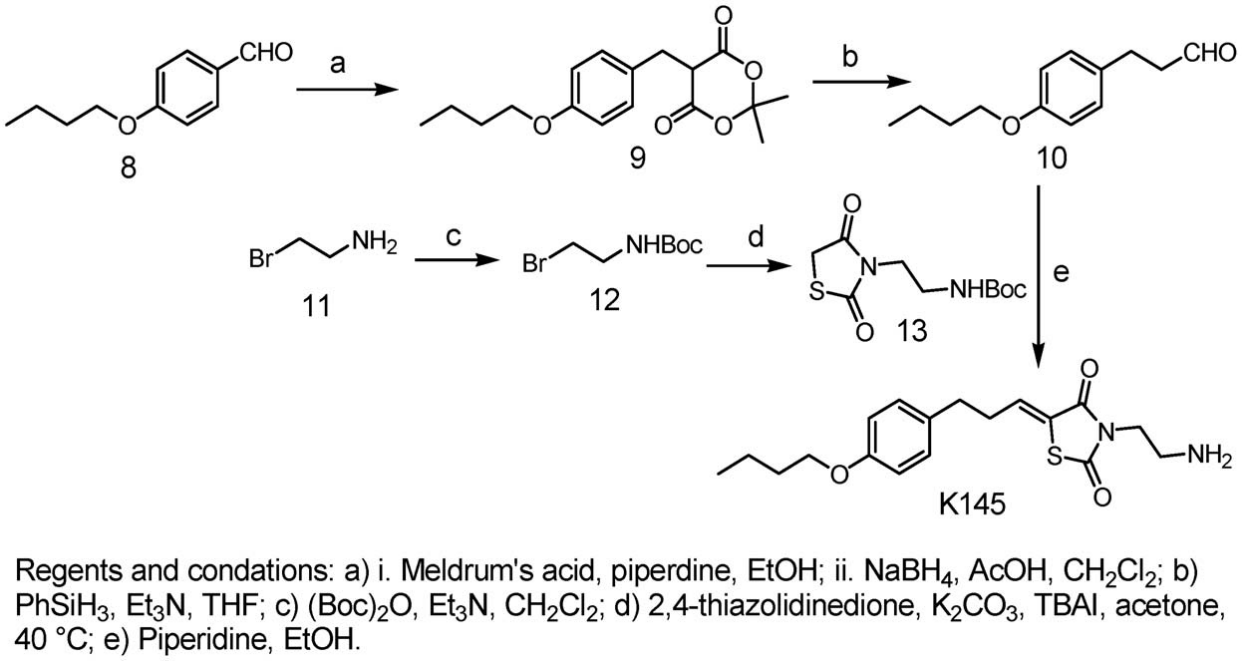
Chemical synthesis of K145.

The synthesis of **K145** is shown in Figure 3. Briefly, 3-(4- butoxy-phenyl)-propionaldehyde (**10**) was prepared from 4-butoxy-benzaldehyde (**8**) by reacting with Meldrum’s acid in the presence of piperidine followed by reduction [29]. Then, the condensation reaction of **10** with **13**, prepared from thiazolidine-2,4-dione, followed by the removal of the Boc protecting group afforded **K145** in good yield.

### *In vitro* Studies

**Figure 4:**
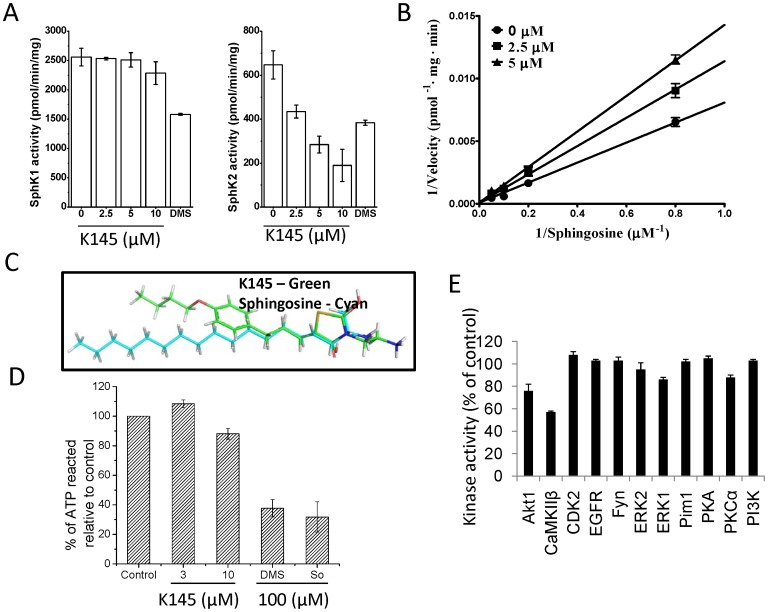
K145 inhibits SphK2 but not SphK1. A) SphK1 and SphK2 activities were measured with 5 µM sphingosine in the absence or presence of the indicated concentrations of **K145** or 10 µM DMS. Data are expressed as percentage SphK activity in the absence of inhibitor; B) SphK2 activity was measured with increasing concentrations of sphingosine and the indicated concentrations of **K145**. Lineweaver-Burk analysis revealed a Vmax of 10820±210 pmol/min per mg of protein, and a K_i_ of 6.4±0.7 µM for SphK2; C) Overlay of **K145** with sphingosine; D) CERK activities were measured with 12.5 µM ceramide in the absence or presence of the indicated concentrations of **K145** or 100 µM of DMS and sphingosine; E) Effect of **K145** (10 µM) on activity of the indicated enzymes was tested by SelectScreen Kinase Profiling from Invitrogen. Data are expressed as percentage of control activity averaged from 2 independent experiments. Data are expressed as mean value ± SEM.

After synthesis, we first examined the effects of **K145** on recombinant SphK1 and SphK2. Notably, **K145** inhibited the activity of SphK2 in a dose-dependent manner with an IC_50_ of 4.30±0.06 µM (Figure 4A), while no inhibition of SphK1 at concentrations up to 10 µM was observed. In contrast, and as expected, DMS (10 µM), a non-selective SphK inhibitor, inhibited both SphK1 and SphK2. Thus, **K145** is a selective SphK2 inhibitor. Lineweaver-Burk analysis revealed a K_i_ of 6.4±0.7 µM for SphK2 and indicated that **K145** is a substrate competitive inhibitor (with sphingosine) (Figure 4B). The overlay of **K145** with sphingosine also supports these results (Figure 4C). Since K145 is a sphingosine analogue, we then tested it against ceramide kinase (CERK) to confirm selectivity. As shown in Figure 4D, at a concentration up to 10 µM, no significant inhibition of CERK was observed. Further screening against eleven other protein kinases (Figure 4E) also demonstrated the relative selectivity of **K145** to SphK2.

**Figure 5:**
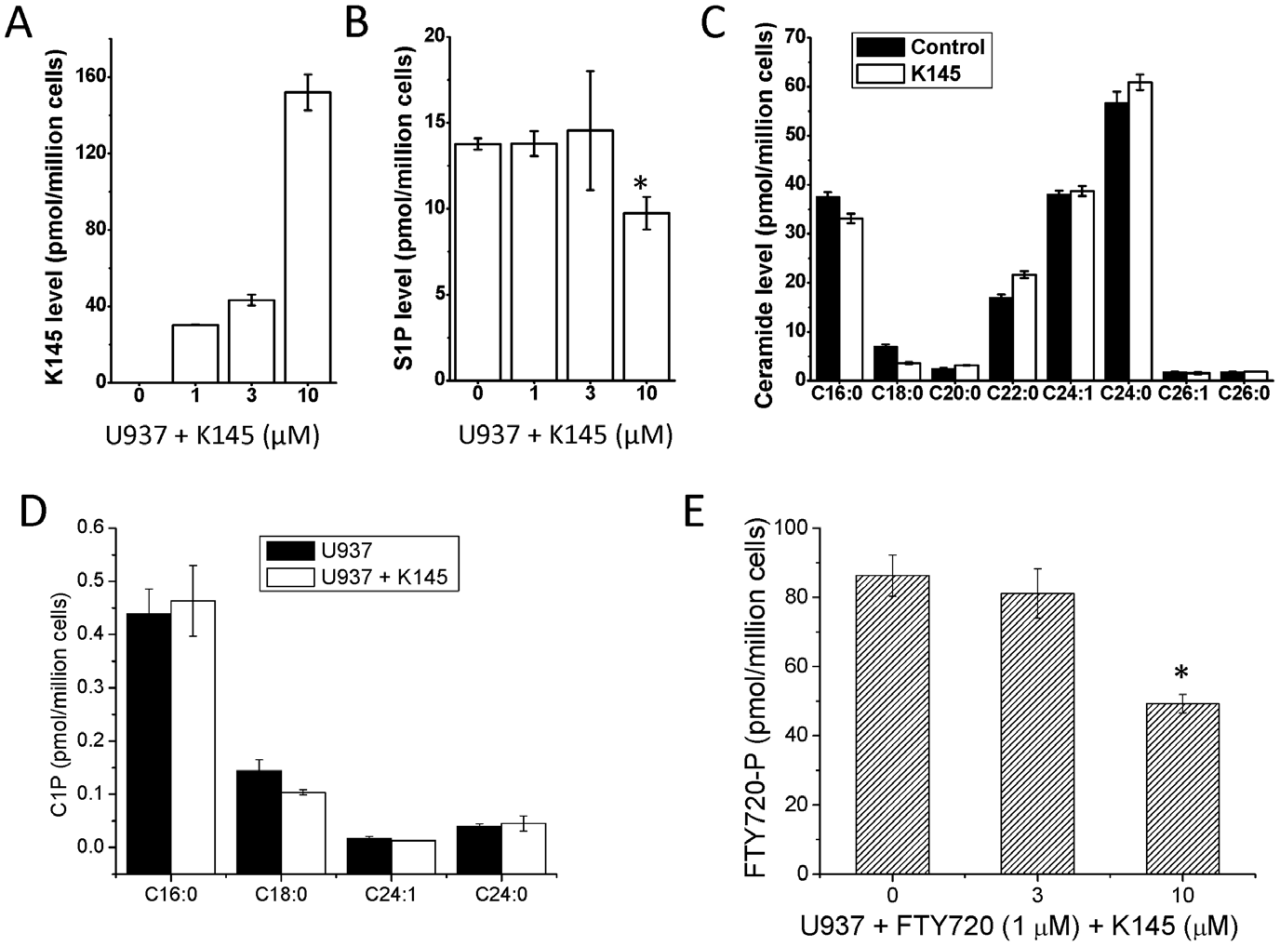
K145 accumulates and suppresses the S1P level. A and B) U937 cells were treated with **K145** at the indicated concentrations for 3 h and the levels of **K145** and S1P were measured by ESI-MS/MS. C) HEK293 cells were treated with K145 (10 µM) for 2 h. Lipids were extracted and different chain length species of ceramide were determined by LC-ESI-MS/MS. Numbers indicate chain length followed by the number of double bonds in the fatty acid. Data are averages of triplicate determinations and are expressed as pmol lipid/10^6^ cells. D) U937 cells were treated with or without **K145** (10 µM) for 3 h and levels of C1P species were determined by ESI-MS/MS. E) U937 cells were treated with FTY720 (1 µM) in the absence or presence of indicated **K145** for 3 h, then FTY720-P was measured by ESI-MS/MS. *P<0.05 compared to control.

**Figure 6:**
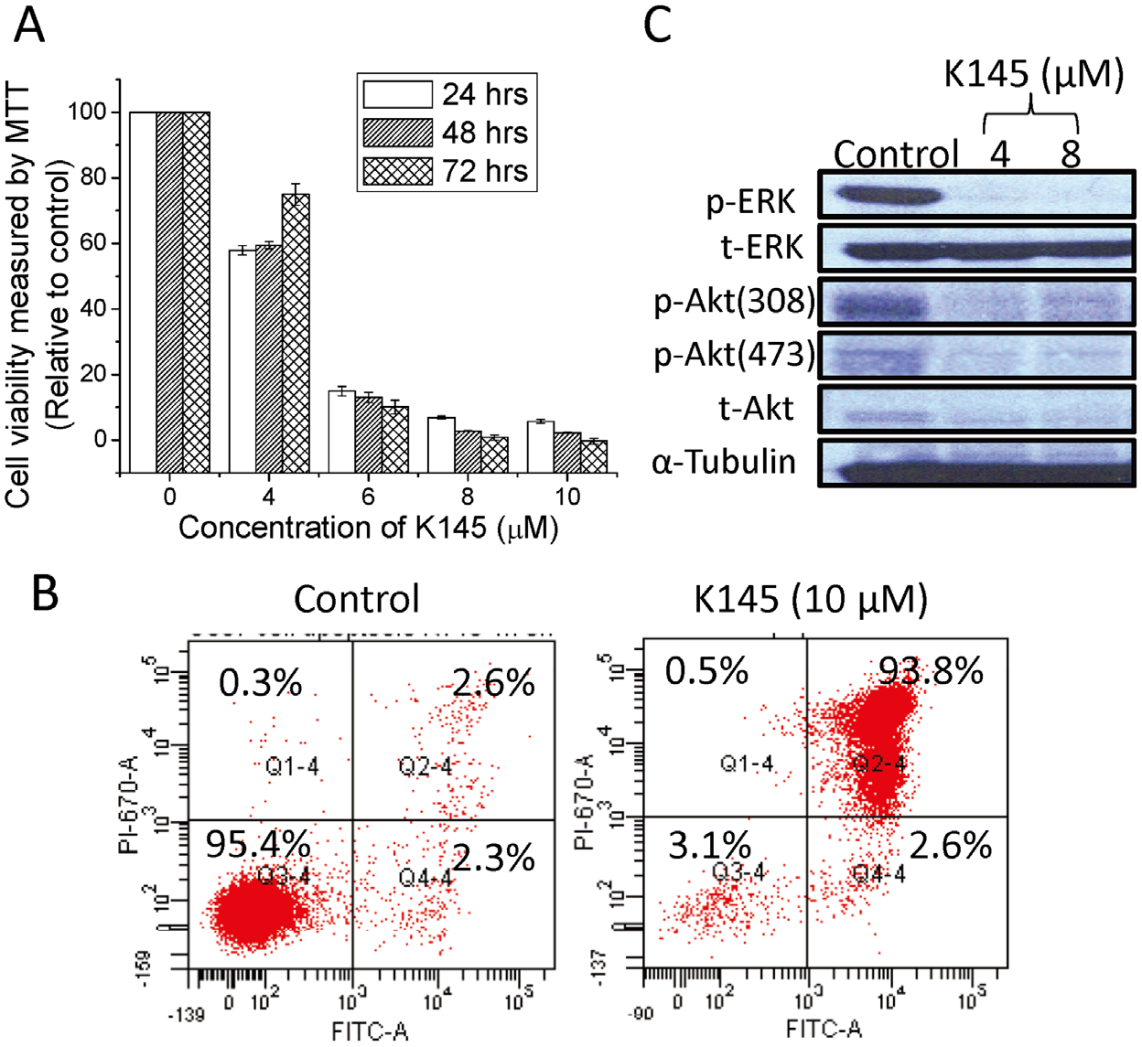
K145 exhibits antiproliferative and apoptotic effects in U937 cells. A) U937 cells were treated with **K145** at indicated concentrations for indicated intervals, the cell viability was determined by MTT assay; B) U937 cells were treated with **K145** (10 µM) for 24 h, then the cells were stained with Annexin V/PI and analyzed by flow cytometry; C) U937 cells were treated with **K145** at indicated concentrations for 3 h, after which cell lysates were analyzed by Western blot using corresponding primary antibodies. The image of Western blot represents the results from one of two independent experiments. Data are expressed as mean value ± SEM.

Next we examined whether K145 affects cellular levels of S1P. Human leukemia U937 cells have been demonstrated to be a good model to test compounds that interfere with the SphK/S1P system and it has previously been shown that S1P is protective against apoptosis of U937 cells [9], [23], [24], [34]. Therefore, we further characterized **K145** in U937 cells. As shown in Figure 5A, **K** **145** is readily taken up by U937 cells in a concentration dependent manner. In agreement with its ability to inhibit SphK2, but not ceramide kinase (Figure 4D), as shown in Figures 5B and 5C, treatment with **K145** (10 µM) caused a decrease of total cellular S1P without significant effects on ceramide levels. The inhibitory potency of **K145** on cellular level of S1P is somewhat less than its IC_50_ (4.3 µM) determined at recombinant SphK2. This might be due to the fact that many enzymes such as SphK1, SphK2, S1P lyase, and S1P phosphatise, not just SphK2, are involved in the regulation of cellular S1P. The level of ceramide-1-phosphate (C1P) was not significantly affected upon treatment with **K145** (10 µM, Figure 5D), which indicates that **K145** does not interfere with CERK and/or ceramide synthase, consistent with the results from recombinant CERK studies. To further confirm its SphK2 selectivity, we then tested the effects of **K145** on the phosphorylation of FTY720, a SphK2 specific substrate [35]. As shown in Figure 5E, **K** **145** inhibited the phosphorylation of FTY720, which further indicates the SphK2 specificity of **K145**. SphK2 inhibitors have been shown to have anti-proliferative activities in cancer models both *in vitro* and *in vivo* [19], [20]. SphK2 has also been recently shown to play a role in mitochondria function and apoptosis. Therefore, we tested the anti-proliferative and apoptotic effects of **K145** in U937 cells. As shown in Figure 6A, **K** **145** significantly inhibited the growth of U937 cells cultured in the presence of 10% serum in a concentration-dependent manner. In agreement with our study, others using a different SphK2 inhibitor ABC294660 [20], or downregulation of SphK2 [15] also observed effects on cell proliferation, while treatment with another SphK2 inhibitor SLR08081 caused only a small decrease in cell viability [36]. **K145** also significantly induced apoptosis in U937 cells under these experimental conditions (Figure 6B). Notably, **K145** induced mainly late apoptosis with a very small percentage of necrotic cells after 24 h treatment in the presence of 10% serum. In contrast, the SphK1 inhibitor SK1-I mainly induced early apoptosis of U937 cells after similar treatment (24 h in the presence of 10% serum) [24]. This suggests that SphK1 and SphK2 may have different roles in the regulation of apoptosis, at least for U937 cells.

S1P has been shown to activate the downstream ERK and Akt signaling pathways [37], [38] and these two signalling cascades have been demonstrated to cooperatively link with each other to regulate apoptosis and the survival of transformed cells including human leukaemia cells [39]. As shown in Figure 6C, phosphorylated ERK and Akt were decreased by treatment of U937 cells with **K145**, consistent with our previous results with TZD analogues as dual-pathway inhibitors [28], [29]. Notably, the inhibition of these two signaling pathways is evident at as low as 4 µM of **K145**, consistent with its IC_50_ values of SphK2 inhibition and anti-proliferation of U937 cells. Studies have demonstrated the synergistic effects in triggering cancer cell death by concomitant interruption of these two pathways, both *in vitro* and *in vivo* using a combination regimen [40]. The simultaneously inhibitory effects of **K145** on ERK and Akt pathways upon its inhibition of SphK2 may explain its significant anti-proliferative and apoptotic effects in U937 cells.

### Molecular Modeling

To further understand the SphK2 selectivity of **K145**, we conducted molecular modeling studies to identify the structural features of **K145** that interact with key residues of SphK2. Since no crystal structure is currently available for either SphK1 or SphK2, we generated structural models of SphK1 and SphK2 using MODELLER [41]. a comparative protein structure modeling program, using the structure of Diacylglycerol kinase from *Bascillus anthracis* str. Sterne (PDB ID: 3T5P) as a template [42], [43]. The overall structural geometries of the resulting models were checked using MOLPROBITY [44] (clash scores for both proteins >97^th^ percentile) and the Ramachandran plots for the backbone conformations showed >96% of residues in allowed regions for both isoforms. Despite low overall homology to the template (∼46% in aligned regions for both isoforms), there is considerable sequence and structural similarity at the sphingosine-binding (C4) domain [45], and we believe that these models provide valuable structural information.

The final optimized models for both proteins were validated with a panel of inhibitors including the reported SphK2 inhibitors shown in Figure 1, a SphK1 selective inhibitor, SK1-I [24], and FTY720, a compound known to bind to both SphKs [46], [47]. The binding sites for SphK2 and SphK1 were defined to encompass all atoms within 20 Å of the CA of Asp344 and Asp178, respectively. Docked poses were generated using GOLD [48] v5.1 and rescored using HINT [49]. The best scoring docked positions for each ligand were selected for minimization (2500 iterations, termination gradient of 0.005 kcal/mol-Å). As shown in Table 1, the relative ordering of HINT scores (H_TOT_) are largely in concordance with the reported binding/inhibitory observations (Table 1). [50], [24]–[27].

**Table 1:** HINT scores of the docked molecules into SphK1 and SphK2.

	HINT Scores^a^	
Ligand	SphK2	SphK1
K145	3011	1506
FTY720	2751	2347
(R)-FTY720-OMe	1878	138
SG-12	1876	1626
ABC294640	153	−73
Trans-12b	218	−985
SK1-I	679	2080

a Previous studies have shown that ∼515 score units correspond to ΔΔG = −1.0 kcal/mol [49]. In the absence of a reference point from a calibration for this specific biomolecular system, the HINT score *differences* between ligands and/or between SphK1 and SphK2 are more meaningful than their specific values.

**Figure 7:**
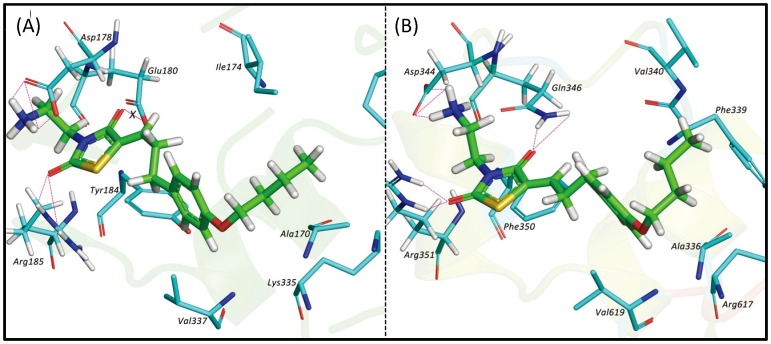
Docking results of K145 to SphKs. Binding mode of K145 in SphK1 (A) and SphK2 (B). **K145** is shown as sticks with carbon in green, while the interacting residues of both kinases are shown as sticks with carbons depicted in cyan. For simplicity, hydrogens are only shown on residues forming hydrogen-bonding interactions with **K145**.

We then docked **K145** to the two kinase models. The docking results revealed that **K145** binds preferentially to SphK2 (Table 1). Specifically, as shown in Figure 7, our model indicates that the terminal –NH_2_ of **K145** forms strong H-bond interactions with the carboxylate group of Asp344 (the putative sphingosine recognizing residue). Other favourable hydrogen bonding interactions are also formed between the guanidino-group of Arg351 and Gln346 with the carbonyl oxygens of the TZD heterocycle. The TZD ring of **K145** shows favourable π-stacking interactions with Phe350 and the 4-butoxy-phenyl ring of **K145** fits into a hydrophobic pocket that consists of Ala336, Val340, Val343, Arg617 and Val619. **K145** showed a very similar binding mode within the sphingosine-binding pocket of SphK1 except that, in contrast to its binding mode in SphK2, the carbonyl oxygen at the 4-position of the TZD ring (interacting with Gln346 in SphK2) showed unfavourable base/base interactions with the carboxylate group of Glu180 (corresponding to SphK2/Gln346). Judging from the sequence similarity in the sphingosine-binding domains of both isoforms, the Gln **→**Glu change in SphK1 is the only significant difference in this region and might be the reason for **K145** showing selectivity towards SphK2.

### *In vivo* Studies

**Figure 8:**
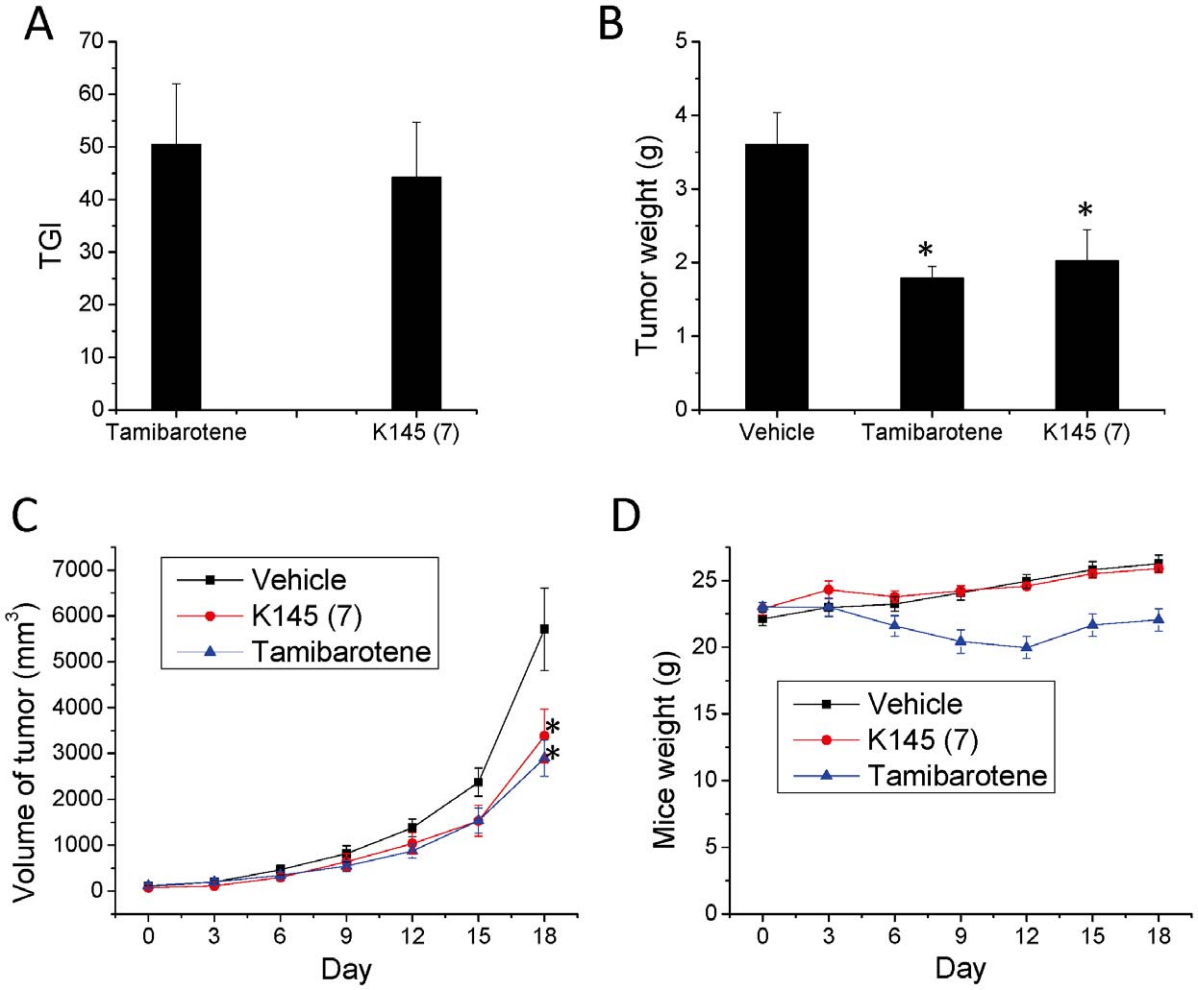
K145 suppresses the growth of U937 xenograft in nude mice. BALB/c-nu mice (n = 7) with palpable U937 xenograft were treated daily with vehicle, tamibarotene (15 mg/kg), or **K145** (15 mg/kg) for 17 days by i.p. injection. A) After treatment, animals were sacrificed and tumors were removed and weighed and the TGI was calculated; B) Tumor volumes were measured every other day during the treatment course; C) Animal weights were measured every other day during treatment course. Data are expressed as mean value ± SD. *P<0.05 compared to control group.

Downregulation of SphK2 or application of a SphK2 inhibitor have shown anti-tumor effects both *in vitro* and *in vivo* [15]–[20] and we have shown the anti-proliferative and apoptotic effects of **K145** in U937 cells; thus, we evaluated the ability of **K145** to inhibit the growth of U937 tumors in nude mice. Tamibarotene [51], a retinoid compound that has been approved for acute promyelocytic leukemia in Japan, was used as a positive control. Both tamibarotene and **K145** were given at a 15 mg/kg dose by i.p. injection. As shown in Figure 8A, **K** **145** significantly inhibited the growth of U937 tumors in nude mice with a TGI of 44.2%, slightly less potent than tamibarotene (TGI = 50.4%) after 17 days treatment. This is also reflected by the tumor weights of treatment groups (Figure 8B). The tumor growth curve during the treatment course (Figure 8C) also attests to the anti-tumor effects of **K145** in this model. Lastly, as shown in Figure 8D, the body weights of **K145**-treated mice remained the same as that of vehicle-treated mice, while tamibarotene treatment caused body weight decreases in the mice. These results strongly suggest that **K145** exhibits comparable *in vivo* anti-tumor activity to tamibarotene, while concomitantly exhibiting less toxicity in this U937 xenograft cancer model.

**Figure 9:**
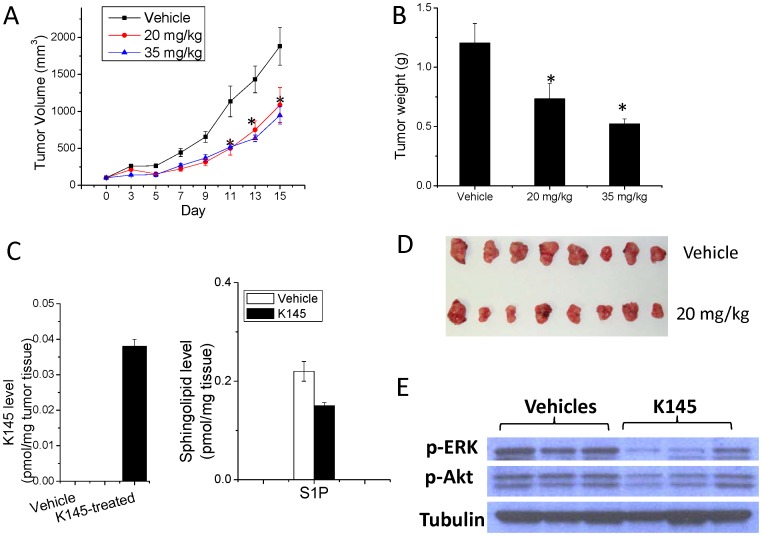
K145 suppresses the growth of JC xenograft in BALB/c mice. BALB/c mice (n = 8) with palpable JC xenograft were treated daily with vehicle or **K145** (20 mg/kg and 35 mg/kg) for 15 days by i.p. injection. A) Tumor volumes were measured every other day; B) After treatment, animals were sacrificed and tumors were removed and weighed; C) The S1P and K145 levels in the tumor samples from vehicle and treatment (20 mg/kg) groups (n = 4) were measured by ESI-MS/MS; D) Images of tumor samples from control and treatment groups (n = 7 for each group) after the experiments; E) Tumor samples (20 mg/kg and control groups) were analyzed by Western blot. Data are expressed as mean value ± SEM. *P<0.05 compared to control group.

On the basis of this *in vivo* anti-tumor activity in nude mice, we attempted to further confirm **K145**’s *in vivo* anti-tumor activity using a syngeneic breast cancer model. In this regard, we selected a tumor model that uses the mouse JC mammary adenocarcinoma cell line growing subcutaneously in immunocompetent BALB/c mice [19], [52]. Since **K145** demonstrated *in vivo* activities at 15 mg/kg dose in the nude mice experiment, we used two doses, 20 mg/kg and 35 mg/kg through i.p. injection in this model. As illustrated in Figure 9A, treatment of BALB/c mice (n = 8) bearing the JC xenograft significantly inhibited tumor growth at both doses with the higher dose being more potent. After 15 days treatment, the mean volume of the JC tumors in the treated-mice at both doses was >50% smaller than that in the vehicle-treated mice. Tumor weights of **K145**-treated mice were also significantly less than that in vehicle-treated mice in a dose-dependent manner (Figure 9B). Post-experiment visual evaluation of the tumor samples also confirms the results (Figure 9D). We analyzed the tumor samples to detect **K145**, the change of S1P by ESI-MS/MS and the change of signaling pathways by Western blot. As shown in Figure 9C, **K** **145** was detected in JC tumors and the S1P level was suppressed compared to vehicle, consistent with the results from U937 cells assays. Notably, the p-ERK and p-Akt levels were decreased in the tumor samples compared to the vehicle controls (Figure 9E), which is consistent with the results from U937 cells (Figure 6C). We did not observe significant changes in body weights and the major organs, such as heart, lung, liver and kidney (data not shown), thus indicating a lack of general toxicity of **K145**.

**Figure 10:**
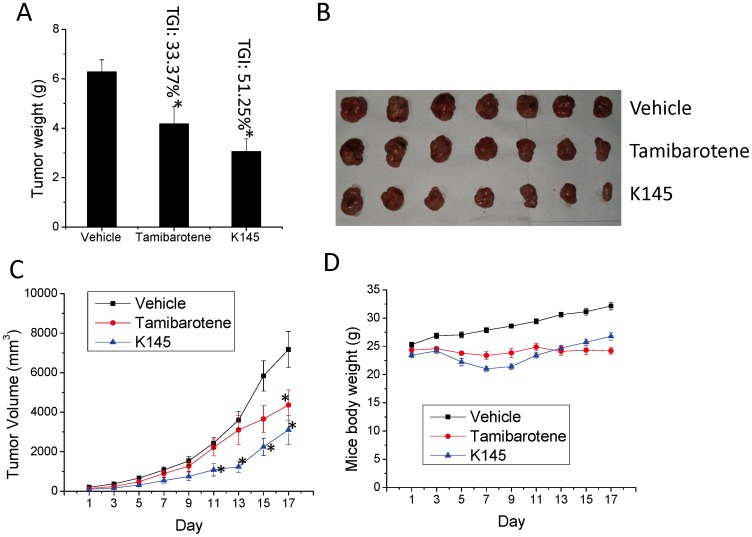
K145 suppresses the growth of U937 tumors in nude mice by oral administration. BALB/c-nu mice (n = 7) with palpable U937 xenograft were treated daily with vehicle, tamibarotene (20 mg/kg), or **K145** (50 mg/kg) for 15 days by oral gavage. After treatment, animals were sacrificed and tumors were removed, weighed and images were taken. A) Tumor weight and TGI comparison; B) Images of tumor samples from control and treatment groups (n = 7 for each group) after the experiments; C) Tumor volumes were measured every other day; D) Animal weights were measured every other day. Data are expressed as mean value ± SEM. *P<0.05 compared to control group.

Lastly, we examined the anticancer activity of K145 to inhibit the tumor growth of U937 cells in nude mice (BALB/c-nu) by oral administration to investigate whether it is orally available. In this experiment, **K145** was given at 50 mg/kg by oral gavage daily for 15 days and tumor volume and animal weights were measured every other day. Again, tamibarotene (20 mg/kg) was used as positive control. As shown in Figure 10A, tumor weights of **K145**-treated mice were significantly less than that in vehicle-treated mice and **K145** exhibited better antitumor activity than tamibarotene at tested doses by oral administration (TGI for K145 and tamibarotene are 51.25% and 33.37%, respectively). Visual examination of the tumor samples also confirmed the significant inhibition of U937 tumor growth by **K145** (Figure 10B). Tumor growth curve also demonstrated the superior anti-tumour activity of **K145** in these experimental settings (Figure 10C). As shown in Figure 10D, at the beginning of the treatment, there was a slight decrease of body weights in **K145**-treated group but the body weights of this group came back in the remaining course of the study. Collectively, the results of *in vivo* studies with **K145** by oral administration demonstrated that **K145** is orally available to inhibit the growth of U937 tumors at 50 mg/kg dose and no apparent toxicity was observed, which is consistent with the results from in vivo studies by i.p. injection administration. To summarize, all *in vivo* results we have obtained strongly suggest that **K145** has *in vivo* anti-tumor activity, thus it may serve as a good lead compound for development of more potent and selective SphK2 inhibitors and new anticancer agents.

### Conclusions

**K145** was identified as a selective SphK2 inhibitor. Biochemical assays using recombinant SphK1 and SphK2 established that **K145** selectively inhibited SphK2 but not SphK1. Molecular modeling and docking studies also suggested that **K145** favorably binds to SphK2 but not SphK1 within their respective sphingosine binding pockets, consistent with the biochemical assay results. Further biological characterization using human leukemia U937 cells demonstrated that **K145** accumulated in U937 cells and inhibited the phosphorylation of FTY720. Furthermore, K145 inhibited the growth of U937 cells, mainly through apoptotic effects. It is well documented that the activation of SphK/S1P system leads to the activation of survival signaling pathways including the ERK and Akt cascades. Moreover, like **K145**, TZD analogues are known to be dual-pathway inhibitors [28], [29]. Consistent with this notion, **K145** significantly inhibited the phosphorylation of ERK and Akt upon treatment of U937 cells and the deactivation of ERK and Akt might contribute to its apoptotic effects as these two signalling pathways have been closely linked to apoptosis in leukemia cells. Most importantly, **K145** was shown to significantly suppress the growth of U937 tumors in nude mice through both intraperitoneal and oral administration without apparent toxicity, thus attesting to its oral bioavailability and *in vivo* anti-tumor actiivty. The use of a syngeneic tumor model further confirmed that **K145** significantly inhibited the growth of JC tumor cells in BALB/c mice at both 20 and 35 mg/kg doses, again without apparent toxicity. These results encourage further optimization of **K145** as a novel lead compound to develop more potent and selective SphK2 inhibitors. Our results may also suggest that development of novel thiazolidine-based inhibitors may provide effective compounds as anticancer agents.

## Materials and Methods

### Chemistry

Reagents and solvents were obtained from commercial suppliers and used as received unless otherwise indicated. All reactions were carried out under inert atmosphere (N_2_) unless otherwise noted. Reactions were monitored by thin-layer chromatography (TLC) (precoated silica gel 60 F_254_ plates, EMD Chemicals) and visualized with UV light or by treatment with Phosphomolybdic acid (PMA). Flash chromatography was performed on silica gel (200–300 mesh, Fisher Scientific) using solvents as indicated. ^1^HNMR and ^13^CNMR spectra were routinely recorded on Bruker ARX 400 spectrometer. The NMR solvent used was CDCl_3_ or DMSO-*d*6 as indicated. Tetramethylsilane (TMS) was used as internal standard. The purity of target compound was determined by elemental analysis.

#### 5-(4-butoxybenzyl)-2,2-dimethyl-1,3-dioxane-4,6-dione (10)

To a stirred solution of 4-butoxybenzaldehyde (891.0 mg, 5.0 mmol) and Meldrum’s acid (720.0 mg, 5.0 mmol) in EtOH (5.0 mL) was added piperidine (two drops). The resulting solution was stirred at room temperature overnight and suction filtration gave a light yellow solid (731.0 mg). To the mixture of the yellow solid and acetic acid (3.0 mL) in CH_2_Cl_2_ (20.0 mL) was added sodium borohydride (314.0 mg, 8.4 mmol) at 0°C. The resulting solution was stirred at room temperature for 1 h, diluted with CH_2_Cl_2_ (30.0 mL) and washed with brine and water. The organic layer was then dried over anhydrous Na_2_SO_4_. After filtration, the mixture was concentrated under vacuum and the residue was purified by flash column chromatography (hexane/acetone = 5/2) to give 10 as a light yellow solid, 28% yield. ^1^H NMR (400 MHz, CDCl_3_): 7.23–7.21 (d, *J = *8.6 Hz, 2H), 6.82–6.79 (d, *J = *8.6 Hz, 2H), 3.92 (t, *J = *6.5 Hz, 2H), 3.71 (t, *J = *4.8 Hz, 1H), 3.44–3.43 (d, *J = *4.8 Hz, 2H), 1.76–1.70 (m, 5H), 1.50–1.44 (m, 5H), 0.96 (t, *J = *7.4 Hz, 3H).

#### 4-butoxyphenyl-3-propanal (11)

To a solution of compound 10 (368.0 mg, 1.2 mmol) in THF (7.0 mL) was added Et_3_N (0.3 mL, 2.4 mmol) followed by phenylsilane (0.4 mL, 3.6 mmol). The resulting solution was stirred for 2 h at room temperature. Water was added to the solution and stirred for 15 min. The reaction mixture was diluted with diethyl ether (15.0 mL) and washed with water and brine. The organic layer was dried over anhydrous Na_2_SO_4_ overnight. After filtration, the filtrate was concentrated and the residue was purified by flash column chromatography (hexane/acetone = 10/1) to give 11 as a light colorless oil, 81% yield. ^1^H NMR (400 MHz, CDCl_3_): 9.81 (t, *J = *1.5 Hz, 1H), 7.10–7.08 (d, *J = *8.6 Hz, 2H), 6.83–6.81 (d, *J = *8.6 Hz, 2H), 3.93 (t, *J = *6.5 Hz, 2H), 2.90 (t, *J = *7.5 Hz, 1H), 2.76–2.72 (m, 2H), 1.77–1.71 (m, 2H), 1.55–1.43 (m, 2H), 0.97 (t, *J = *7.4 Hz, 3H).

#### *t*-butyl 2-bromoethylcarbamate (13)

To a stirred suspension of bromoethylamine hydrobromide (20.5 g, 100.0 mmol), (Boc)_2_O (21.8 g, 100.0 mmol) in CH_2_Cl_2_ (200.0 mL) was added triethylamine (13.9 mL, 100.0 mmol) dropwise at 0°C. After addition, the mixture was stirred at room temperature overnight, and then water was added. The separated CH_2_Cl_2_ layer was washed with brine, and dried over anhydrous Na_2_SO_4_. Removal of the solvents gave 13 as colorless oil, 89% yield. ^1^H NMR (400 MHz, CDCl_3_): δ 4.95 (s, 1H), 3.54–3.53 (m, 2H), 3.47–3.45 (m, 2H), 1.45(s, 9H).

#### *t*-Butyl 2-(2,4-dioxothiazolidin-3-yl)-ethylcarbamate (14)

A mixture of thiazolidine-2,4-dione (7.9 g, 68.0 mmol), 13 (17.9 g, 80.0 mmol), K_2_CO_3_ (11.1 g, 92.0 mmol), and TBAI (2.5 g, 6.8 mmol) in acetone (100.0 mL) was stirred at 40°C for 10 h. The reaction mixture was cooled to room temperature and filtered through a short bed of celite. The filtrate was concentrated under vacuum and the residue was purified by flash chromatography (Hexane/EtOAc = 4/1 to 2/1) to give 14 as white solid, 70% yield. ^1^H NMR (300 MHz, CDCl_3_): δ 3.94 (s, 2H), 3.78–3.74 (t, *J = *6.0 Hz, 2H), 3.40–3.34 (t, *J = *6.0 Hz, 2H), 1.43(s, 9H).

#### 3-(2-aminoethyl)-5-(3-(4-butoxyphenyl)propylidene)thiazolidine-2,4-dione (7)

A solution of 14 (2.0 g, 7.7 mmol), 4-butoxyphenyl-3-propanal (1.9 g, 7.7 mmol) and piperidine (0.2 mL, 3.6 mmol) in EtOH (60.0 mL) was stirred at room temperature overnight. The solvents were removed under vacuum and the residue was purified by flash column chromatography (hexane/EA = 8/1) to give *t*-butyl 2-(5-(3-(4-butoxyphenyl)propylidene)-2,4-dioxothiazolidin-3-yl)ethylcarbamate as a white solid, 60% yield. ^1^H NMR (400 MHz, CDCl3): 7.10–7.06 (m, 3H), 6.85–6.80 (d, *J = *8.6 Hz, 2H), 4.72 (s, 1H), 3.94 (t, *J = *6.5 Hz, 2H), 3.81 (t, *J = *5.5 Hz, 2H), 3.39–3.38 (m, 2H), 2.78 (t, *J = *7.4 Hz, 2H)**,** 2.53–2.48 (q, *J = *7.6 Hz, 2H), 1.80–1.72 (m, 2H), 1.49–1.44 (m, 2H), 1.40 (s, 9H), 0.97 (t, *J = *7.4 Hz, 3H). To a stirred solution of *t*-butyl 2-(5-(3-(4-butoxyphenyl)propylidene)-2,4-dioxothiazolidin-3-yl)ethylcarbamate (2.3 g, 4.7 mmol) in EtOAc (20.0 mL) was added 4 M HCl in dioxane (6.0 mL), the solution was stirred at room temperature for 5 h. The precipitate was collected by filtration to give the HCl salt of 7 as white solid, 75% yield. ^1^H NMR (400 MHz, DMSO-*d*_6_): 8.08 (brs, 3H), 7.15–7.13 (d, *J = *8.5 Hz, 2H), 7.01 (t, *J = *7.4 Hz, 1H), 6.86–6.84 (d, *J = *8.5 Hz, 2H), 3.92 (t, *J = *6.4 Hz, 2H), 3.83 (t, *J = *6.0 Hz, 2H), 3.00 (m, 2H), 2.76 (t, *J = *7.3 Hz, 2H), 2.53–2.48 (m, 2H), 1.69–1.64 (m, 2H), 1.45–1.39 (m, 2H), 0.92 (t, *J = *7.3 Hz, 3H); ^13^C NMR (100 MHz, DMSO-*d*_6_): 167.5, 164.4, 157.1, 137.3, 131.9, 129.2, 125.1, 114.3, 66.9, 36.5, 32.9, 32.1, 30.7, 18.6, 13.6. Anal. (C_18_H_25_ClN_2_O_3_S) Calcd. C: 56.17%, H: 6.55%, N: 7.28%, S: 8.33%; found C: 55.91%, H: 6.69%, N: 7.14%, S: 8.16%.

### Homology and Molecular Modeling

Human SphK1 (Accession: Q9NYA1) and SphK2 (Accession: NP_001191088) sequences were obtained from the NCBI database (www.ncbi.nih.gov/protein/). A Position Specific Iterated BLAST [42], [43] search against the database of Protein Data Bank proteins identified a kinase – diacylgycerol kinase from *Bacillus anthracis* str. Sterne (PDB ID: 3T5P), as the closest match to both proteins with ∼25% identity and ∼46% homology in the aligned regions to both isoforms of SphK. Sequence alignments of each SphK1 and SphK2 with 3T5P were performed using CLUSTALX2 [53]. Unaligned regions in both the proteins were deleted. A total of 100 homology models for each isoform were generated based on these alignments, using MODELLER 9v10 [54]. The top 5 models for each kinase with the lowest DOPE (Discrete Optimized Protein Energy) scores and molpdf scores (a MODELLER object function score) and with GA341 [55] scores closest to 1 were chosen for further refinement. The side chains for each model were optimized using SCWRL (dunbrack.fcc.edu/scwrl4/). Hydrogens were added to these top models using SYBYL 8.1 (www.tripos.com) and subsequently subjected to Powell minimization for 10000 iterations in the Tripos force field with a 0.005 kcal/mol-Å termination gradient. The quality of minimized models was evaluated using MOLPROBITY (molprobity.biochem.duke.edu/), which performs an all-atom contact analysis to give a ‘clashscore’ that is indicative of the number of serious clashes (>0.4 Å) per 1000 atoms. Poor side-chain rotamers and unreasonable bond lengths and angles were checked. Ramachandran plots were also generated using MOLPROBITY to check the backbone-geometry of the models. Atom clashes and bad bond lengths and angles were optimized with further minimization. Sphingosine, the natural substrate for both kinases, was docked into the C4 domain (putative Sph binding domain, Leu163– Phe197 for SphK1 and Cys329– Val363 for SphK2 [56]) of each model, using GOLD v5.1 [48]. The docked poses were scored using HINT [49].

The “best” model of both SphK1 and SphK2 was then chosen based on its overall stability and its ability to accommodate Sphingosine in its C4 domain. Model-044 was the best model for SphK1, with a clashscore of 3.2 (97^th^ percentile) and with 96.8% residues in allowed regions on Ramachandran plots. Model-055 was the best model for SphK2, with a clashscore of 2.03 (99^th^ percentile) and with 98.5% residues in allowed regions on Ramachandran plots.

The optimized models of both SphK1 and SphK2 were used for the docking studies. The structures of inhibitors were sketched using SYBYL v8.1, and subjected to minimization to obtain low energy structures. The docking simulations were performed using GOLD v5.1. The binding site was defined to encompass all atoms within 20 Å of CA of Asp178 of SphK1 (Asp344 of SphK2). Fifty solutions for each inhibitor molecule were generated with a protein hydrogen-bond constraint that the carboxylate of Asp178 of SphK1 (Asp344 of SphK2) forms a hydrogen bond with ligand, since the Asp is important for recognition of Sphingosine [48]. The docked poses were scored using HINT. The poses with the best HINT scores were complexed with the protein and the protein-ligand complex was subjected to minimization, to remove steric clashes and get an induced-fit model. The binding modes of the ligands after minimization were re-scored using HINT.

### Cell Culture

Human leukemia U937 cells and murine JC mammary cells were obtained from American Type Culture Collection (ATCC, Manassas, VA) and cultured in RPMI 1640 medium (Life Technologies, Inc., Grand Island, NY) supplemented with 10% (v/v) of heat-inactivated fetal bovine serum (FBS, Hyclone, Logan, UT). Cells were maintained at 37°C in a fully humidified atmosphere containing 5% CO_2_. All reagents were prepared and used as recommended by their suppliers.

### SphK Activity

Lysates from cells overexpressing SphK1 or SphK2 were used for determining the effects of inhibitors on enzyme activity [11]. SphK1 activity was determined in the presence of 5 µM sphingosine and [γ-^32^P]ATP (10 µCi, 1 mM) containing MgCl_2_ (10 mM) in 0.25% Triton X-100, which inhibits SphK2, as described previously [11]. SphK2 activity was determined with sphingosine added as a complex with 4 mg/ml BSA and [γ-^32^P]ATP in the presence of 1 M KCl, conditions in which SphK2 activity is optimal and SphK1 strongly inhibited [18].

### Ceramide Kinase Assay

Ceramide and human recombinant ceramide kinase (CERK) were obtained from BPS bioscience, Kinase-Glo luminescent assay regents were obtained from Promega. Assays were carried out on 96 wells plate at 50 µL scale in triplicate. For each well, compounds, 15 µM ceramide (prepared freshly according to the manufacturer’s instruction), and 40 ng CERK were incubated at RT for 5 min, the reaction was started by addition of ATP (5 µM final concentration). Incubated at 37°C for 25 min, 50 µL of Kinase-Glo luminescent regent was added and incubated at RT for 10 min. The luminescence was recorded by a FlexStation 3 plate reader (Molecular Devices, CA).

### Lipidomics Analysis

Four million U937 cells were treated by K145 for 3 h. Cells were collected and washed with PBS and analyzed by ESI-MS/MS. For influence of FTY720-P formation, 4 million U937 cells were incubated with K145 together with 1 µM FTY720 for 3 h, then cells was collected and washed with PBS and analyzed by LC-MS/MS as described previously [57].

### Cell Proliferation Assays

U937 cells (20,000 cells/well) were plated into 96-well plate and treated with K145 for 72 h. Then 10 µL of MTT [3-(4,5-Dimethylthiazol-2-yl)-2,5-diphenyltetrazolium bromide 5 mg/mL in PBS] was added and cells incubated for an additional 2 h at 37°C in a fully humidified atmosphere containing 5% CO_2_. After centrifuge, medium (170 µL) was removed and DMSO (100 µL) was added to each well. The absorbance was read by a FlexStation 3 plate reader (Molecular Devices, CA) at a wavelength of 570 nm. Values were expressed as a percentage relative to those obtained in untreated controls.

### Apoptosis Assay

U937 (1×10^6^) cells were treated with K145 at indicated concentrations and indicated intervals, then cells were collected and washed twice with cold PBS. Cells were resuspended in buffer containing 10 mM HEPES [N-2-hydroxyethylpiperazine-N’-2-ethanesulfonic acid]/NaOH, pH 7.4, 140 mM NaOH, and 2.5 mM CaCl_2_. The cells were incubated with annexin V-fluorescein isothiocyanate (FITC) (BD PharMingen, San Diego, CA) and propidium iodide (PI) for 15 min at room temperature per manufacturer’s instruction. The samples were analyzed by flow cytometry using a Beckton Dickinson FACSCanto machine within 1 h to determine the percentage of cells displaying annexin V-FITC staining (early apoptosis) or both annexin V-FITC and PI staining (late apoptosis).

### Western Blotting

U937 Cells (2×10^5^/mL) were treated with K145 at indicated concentration for 1 h in the presence of 10% FBS. Cells were lysed by sonication in sample buffer [62.5 mM Tris base (pH 6.8), 2% SDS, 50 mM DTT, 10% glycerol, 0.1% bromphenol blue, and 5 mg/mL each chymostatin, leupeptin, aprotinin, pepstatin, and soybean trypsin inhibitor] and boiled for 5 min. For analysis of phospho-proteins, 1 mM of sodium orthovanadate and sodium pyrophosphate was added to the sample buffer. Proteins were collected from the supernatant after centrifugation at 12,800×g for 5 min, and quantified using Coomassie Protein Assay Reagent (Pierce, Rockford, IL). Equal amounts of protein (30.0 µg) were separated by SDS-PAGE on 4–10% tris/glycine gel (Bio-Rad) and electrotransferred onto a PVDF membrane (Bio-Rad). For blotting phosphoproteins, no SDS was included in the transfer buffer. The blots were blocked with 5% nonfat dry milk in TBS-Tween 20 (0.1%) at room temperature for 1 hour and probed with the appropriate dilution of primary antibody overnight at 4°C. The blots were washed twice with TBS-Tween 20 for 15 min and then incubated with a 1∶2000 dilution of horseradish peroxidase-conjugated secondary antibody (Kirkegaard & Perry, Gaithersburg, MD) in 5% nonfat dry milk/TBS-Tween 20 at room temperature for 1 h. After washing twice in TBS-Tween 20 for 15 min, the immunopositive bands were visualized with Western Blot Chemiluminescence Reagent (NEN Life Science Products, Boston, MA). Where indicated, the blots were re-probed with antibodies against GAPDH to ensure equal loading and transfer of proteins. The following antibodies were used as primary antibodies: Cell Signaling, MA: phospho-p44/42 MAPK (Thr202/Tyr204) antibody (1∶1000, rabbit polyclonal), p44/42 MAPK antibody (1∶1000, rabbit polyclonal), phospho-Akt (Ser473) antibody (1∶1000, rabbit polyclonal), Santa Cruz, CA: Akt1/2/3 (H-136) (1∶1000, rabbit polyclonal).

### *In vivo* Antitumor Evaluations

All experiments involving animals were carried out in strict accordance with the recommendations in the Guidelines and Regulations of Institutional Animal Care and Use Committee (IACUC) of the Virginia Commonwealth University (VCU). The protocol was approved by the Committee on the Ethics of Animal Experiments of VCU (IACUC Number: AM10206). For U937 xenografts studies, female BALB/c-nu nude mice (Charles River, NC) were maintained under specific pathogen-free conditions. Human leukemia U937 cells (1 × 10^6^, ATCC, Manassas, VA) in logarithmic growth phase were implanted in the right flanks of each mouse. Once the U937 xenografts reached a palpable size, mice were randomly assigned to control group or treatment groups (n = 7). Compound K145 and Tamibarotene were administered via intraperitoneal (i.p.) injection at 15 mg/kg dose daily for 17 days. For oral administration, K145 and Tamibarotene were administered at 50 mg/kg and 20 mg/kg, respectively, by oral gavage. Body weight and tumor size were measured every three days and the tumor volume was calculated using the equation V = *ab* ^2^/2, where *a* and *b* are the longest and shortest diameters, respectively. At the end of treatment, mice were sacrificed and tumors were collected and weighted. Tumor growth inhibition (TGI) was calculated as TGI = (M_v_ – M_d_)/M_v_ where M_v_ is the mean tumor weight of vehicle-treated group and M_d_ is the mean tumor weight of drug-treated group.

For syngeneic cancer studies, the transformed mouse JC mammary adenocarcinoma cells (1 × 10^6^, ATCC, Manassas, VA) were implanted subcutaneously in immunocompetent BALB/c mice (18–20 g, Charles River, NC). Once the JC xenografts were detectable, mice were then randomly grouped into control or treatment groups (n = 8). K145 was given (20 mg/kg and 35 mg/kg) daily for 15 days. Body weights and volume of tumors were measured every other day, and tumor volume (V) was again calculated. At the end of treatment, mice were sacrificed, tumors were collected and weighted. Tumor samples were homogenized in lysis buffer (50 mM Tris-HCl, 150 mM NaCl, 1 mM EDTA, 1% Triton X100, 0.25% sodium deoxycholate, 0.1% SDS, 1 mM PMSF, pH 7.4) supplemented with protease and phosphatase inhibitor cocktail (Roche) for Western blot analysis.

## References

[1] Spiegel S, Milstien S (2003) Sphingosine-1-phosphate: an enigmatic signalling lipid. Nat Rev Mol Cell Biol 4: 397–407. 12728273 10.1038/nrm1103

[2] Pitson SM (2011) Regulation of sphingosine kinase and sphingolipid signaling. Trends Biochem Sci 36: 97–107. 20870412 10.1016/j.tibs.2010.08.001

[3] Kohama T, Olivera A, Edsall L, Nagiec MM, Dickson R, et al (1998) Molecular cloning and functional characterization of murine sphingosine kinase. J Biol Chem 273: 23722–23728. 9726979 10.1074/jbc.273.37.23722

[4] Liu H, Sugiura M, Nava VE, Edsall LC, Kono K, et al (2000) Molecular cloning and functional characterization of a novel mammalian sphingosine kinase type 2 isoform. J Biol Chem 275: 19513–19520. 10751414 10.1074/jbc.M002759200

[5] Takabe K, Paugh SW, Milstien S, Spiegel S (2008) “Inside-out” signaling of sphingosine-1-phosphate: therapeutic targets. Pharmacol Rev 60: 181–195. 18552276 10.1124/pr.107.07113 PMC2695666

[6] Hait NC, Allegood J, Maceyka M, Strub GM, Harikumar KB, et al (2009) Regulation of histone acetylation in the nucleus by sphingosine-1-phosphate. Science 325: 1254–1257. 19729656 10.1126/science.1176709 PMC2850596

[7] Alvarez SE, Harikumar KB, Hait NC, Allegood J, Strub GM, et al (2010) Sphingosine-1-phosphate is a missing cofactor for the E3 ubiquitin ligase TRAF2. Nature 465: 1084–1088. 20577214 10.1038/nature09128 PMC2946785

[8] Hannun YA, Obeid LM (2008) Principles of bioactive lipid signalling: lessons from sphingolipids. Nat Rev Mol Cell Biol 9: 139–150. 18216770 10.1038/nrm2329

[9] Cuvillier O, Pirianov G, Kleuser B, Vanek PG, Coso OA, et al (1996) Suppression of ceramide-mediated programmed cell death by sphingosine-1-phosphate. Nature 381: 800–803. 8657285 10.1038/381800a0

[10] Pyne S, Bittman R, Pyne NJ (2011) Sphingosine kinase inhibitors and cancer: seeking the golden sword of Hercules. Cancer Res 71: 6576–6582. 21940750 10.1158/0008-5472.CAN-11-2364 PMC3206172

[11] Siow D, Wattenberg B (2011) The compartmentalization and translocation of the sphingosine kinases: mechanisms and functions in cell signaling and sphingolipid metabolism. Crit Rev Biochem Mol Biol 46: 365–375. 21864225 10.3109/10409238.2011.580097 PMC3183286

[12] Johnson KR, Becker KP, Facchinetti MM, Hannun YA, Obeid LM (2002) PKC-dependent activation of sphingosine kinase 1 and translocation to the plasma membrane. Extracellular release of sphingosine-1-phosphate induced by phorbol 12-myristate 13-acetate (PMA). J Biol Chem 277: 35257–35262. 12124383 10.1074/jbc.M203033200

[13] Igarashi N, Okada T, Hayashi S, Fujita T, Jahangeer S, et al (2003) Sphingosine kinase 2 is a nuclear protein and inhibits DNA synthesis. J Biol Chem 278: 46832–46839. 12954646 10.1074/jbc.M306577200

[14] Gault CR, Obeid LM (2011) Still benched on its way to the bedside: sphingosine kinase 1 as an emerging target in cancer chemotherapy. Crit Rev Biochem Mol Biol 46: 342–351. 21787121 10.3109/10409238.2011.597737 PMC3144498

[15] Gao P, Smith CD (2011) Ablation of sphingosine kinase-2 inhibits tumor cell proliferation and migration. Mol Cancer Res 9: 1509–1519. 21896638 10.1158/1541-7786.MCR-11-0336 PMC3219805

[16] Liu H, Toman RE, Goparaju SK, Maceyka M, Nava VE, et al (2003) Sphingosine kinase type 2 is a putative BH3-only protein that induces apoptosis. J Biol Chem 278: 40330–40336. 12835323 10.1074/jbc.M304455200

[17] Van BrocklynJR, Jackson CA, Pearl DK, Kotur MS, Snyder PJ, et al (2005) Sphingosine kinase-1 expression correlates with poor survival of patients with glioblastoma multiforme: roles of sphingosine kinase isoforms in growth of glioblastoma cell lines. J Neuropathol Exp Neurol 64: 695–705. 16106218 10.1097/01.jnen.0000175329.59092.2c

[18] Hait NC, Sarkar S, Le Stunff H, Mikami A, Maceyka M, et al (2005) Role of sphingosine kinase 2 in cell migration toward epidermal growth factor. J Biol Chem 280: 29462–29469. 15951439 10.1074/jbc.M502922200

[19] French KJ, Zhuang Y, Maines LW, Gao P, Wang W, et al (2010) Pharmacology and antitumor activity of ABC294640, a selective inhibitor of sphingosine kinase-2. J Pharmacol Exp Ther 333: 129–139. 20061445 10.1124/jpet.109.163444 PMC2846016

[20] Beljanski V, Lewis CS, Smith CD (2011) Antitumor activity of sphingosine kinase 2 inhibitor ABC294640 and sorafenib in hepatocellular carcinoma xenografts. Cancer Biol Ther 11: 524–534. 21258214 10.4161/cbt.11.5.14677 PMC3087901

[21] Strub GM, Paillard M, Liang J, Gomez L, Allegood JC, et al (2011) Sphingosine-1-phosphate produced by sphingosine kinase 2 in mitochondria interacts with prohibitin 2 to regulate complex IV assembly and respiration. FASEB J 25: 600–612. 20959514 10.1096/fj.10-167502 PMC3023391

[22] French KJ, Upson JJ, Keller SN, Zhuang Y, Yun JK, et al (2006) Antitumor activity of sphingosine kinase inhibitors. J. Pharmacol Exp Ther 318: 596–603. 16632640 10.1124/jpet.106.101345

[23] Kennedy AJ, Mathews TP, Kharel Y, Field SD, Moyer ML, et al (2011) Development of amidine-based sphingosine kinase 1 nanomolar inhibitors and reduction of sphingosine 1-phosphate in human leukemia cells. J Med Chem 54: 3524–3548. 21495716 10.1021/jm2001053 PMC3119570

[24] Paugh SW, Paugh BS, Rahmani M, Kapitonov D, Almenara JA, et al (2008) A selective sphingosine kinase 1 inhibitor integrates multiple molecular therapeutic targets in human leukemia. Blood 112: 1382–1391. 18511810 10.1182/blood-2008-02-138958 PMC2515133

[25] Kim JW, Kim YW, Inagaki Y, Hwang YA, Mitsutake S, et al (2005) Synthesis and evaluation of sphingoid analogs as inhibitors of sphingosine kinases. Bioorg Med Chem 13: 3475–3485. 15848761 10.1016/j.bmc.2005.02.053

[26] Lim KG, Sun C, Bittman R, Pyne NJ, Pyne S (2011) (R)-FTY720 methyl ether is a specific sphingosine kinase 2 inhibitor: Effect on sphingosine kinase 2 expression in HEK 293 cells and actin rearrangement and survival of MCF-7 breast cancer cells. Cell Signal 23: 1590–1595. 21620961 10.1016/j.cellsig.2011.05.010 PMC3148273

[27] Raje MR, Knott K, Kharel Y, Bissel P, Lynch KR, et al (2012) Design, synthesis and biological activity of sphingosine kinase 2 selective inhibitors. Bioorg Med Chem 20: 183–194. 22137932 10.1016/j.bmc.2011.11.011 PMC3748591

[28] Li Q, Al-Ayoubi A, Guo T, Zheng H, Sarkar A, et al (2009) Structure-activity relationship (SAR) studies of 3-(2-amino-ethyl)-5-(4-ethoxy-benzylidene)-thiazolidine-2,4-dione: development of potential substrate-specific ERK1/2 inhibitors. Bioorg Med Chem Lett 19: 6042–6046. 19796943 10.1016/j.bmcl.2009.09.057 PMC6088847

[29] Liu K, Rao W, Parikh H, Li Q, Guo TL, et al (2012) 3,5-Disubstituted-thiazolidine-2,4-dione analogs as anticancer agents: Design, synthesis and biological characterization. Eur J Med Chem 47: 125–137. 22074985 10.1016/j.ejmech.2011.10.031

[30] Baell JB, Holloway GA (2010) New substructure filters for removal of pan assay interference compounds (PAINS) from screening libraries and for their exclusion in bioassays. J Med Chem 53: 2719–2740. 20131845 10.1021/jm901137j

[31] Tomasic T, Masic LP (2009) Rhodanine as a Privileged Scaffold in Drug Discovery. Curr Med Chem 16: 1596–1629. 19442136 10.2174/092986709788186200

[32] Mendgen M, Steuer C, Klein CD (2012) Privileged scaffolds or promiscuous binders: A comprehensive study on rhodanines and related heterocycles in medicinal chemistry. J Med Chem 55: 743–753. 22077389 10.1021/jm201243p

[33] Knight SD, Adams ND, Burgess JL, Chaudhari AM, Darcy MG, et al (2010) Discovery of GSK2126458, a highly potent inhibitor of PI3K and the mammalian target of rapamycin. ACS Med Chem Lett 1: 39–43. 24900173 10.1021/ml900028r PMC4007793

[34] Cuvillier O, Levade T (2001) Sphingosine-1-phosphate antagonizes apoptosis of human leukemia cells by inhibiting release of cytochrome c and Smac/DIABLO from mitochondria. Blood 98: 2828–2836. 11675357 10.1182/blood.v98.9.2828

[35] Paugh SW, Payne SG, Barbour SE, Milstien S, Spiegel S (2003) The immunosuppressant FTY720 is phosphorylated by sphingosine kinase type 2. FEBS Lett 554: 189–193. 14596938 10.1016/s0014-5793(03)01168-2

[36] Kharel Y, Raje M, Gao M, Gellett AM, Tomsig JL, et al (2012) Sphingosine kinase type 2 inhibition elevates circulating sphingosine-1-phosphate. Biochem J 447: 149–157. 22747486 10.1042/BJ20120609 PMC3443596

[37] Shu X, Wu W, Mosteller RD, Broek D (2002) Sphingosine kinase mediates vascular endothelial growth factor-induced activation of ras and mitogen-activated protein kinases. Mol Cell Biol 22: 7758–7768. 12391145 10.1128/MCB.22.22.7758-7768.2002 PMC134718

[38] Osawa Y, Banno Y, Nagaki M, Brenner DA, Naiki T, et al (2001) TNF-α-induced sphingosine-1-phosphate inhibits apoptosis through a phosphatidylinositol-3-kinase/Akt pathway in human hepatocytes. J Immunol 167: 173–180. 11418646 10.4049/jimmunol.167.1.173

[39] Steelman LS, Abrams SL, Whelan J, Bertrand FE, Ludwig DE, et al (2008) Contributions of the Raf/MEK/ERK, PI3K/PTEN/Akt/mTOR and Jak/STAT pathways to leukemia. Leukemia 22: 686–707. 18337767 10.1038/leu.2008.26

[40] Grant.S (2008) Cotargeting survival signaling pathways in cancer J Clin Invest. 118: 3003–3006. 10.1172/JCI36898 PMC2518078 18725993

[41] Sali A, Blundell TL (1993) Comparative protein modelling by satisfaction of spatial restraints, J Mol Biol. 234: 779–815. 10.1006/jmbi.1993.1626 8254673

[42] Altschul SF, Madden TL, Schäffer AA, Zhang J, Zhang Z, et al (1997) Gapped BLAST and PSI-BLAST: a new generation of protein database search programs. Nucleic Acids Res 25: 3389–3402. 9254694 10.1093/nar/25.17.3389 PMC146917

[43] Altschul SF, Wootton JC, Gertz EM, Agarwala R, Morgulis A, et al (2005) Protein database searches using compositionally adjusted substitution matrices., FEBS J. 272: 5101–5109. 10.1111/j.1742-4658.2005.04945.x PMC1343503 16218944

[44] Chen VB, Arendall III WB, Headd JJ, Keedy DA, Immormino RM, et al (2010) MolProbity: all-atom structure validation for macromolecular crystallography. Acta Cystallographica D66: 12–21. 10.1107/S0907444909042073 PMC2803126 20057044

[45] Yokota S, Taniguchi Y, Kihara A, Mitsutake S, Igarashi Y (2004) Asp177 in C4 domain of mouse sphingosine kinase 1a is important for the sphingosine recognition. FEBS Letters 578: 106–110. 15581625 10.1016/j.febslet.2004.10.081

[46] Tonelli F, Lim KG, Loveridge C, Long J, Pitson SM, et al (2010) FTY720 and (S)-FTY720 vinylphosphonate inhibit sphingosine kinase 1 and promote its proteasomal degradation in human pulmonary artery smooth muscle, breast cancer and androgen-independent prostate cancer cells. Cell Signal 22: 1536–1542. 20570726 10.1016/j.cellsig.2010.05.022 PMC2947314

[47] Lim KG, Tonelli F, Li Z, Lu X, Bittman R, et al (2011) FTY720 analogues as sphingosine kinase 1 inhibitors: enzyme inhibition kinetics, allosterism, proteasomal degradation, and actin rearrangement in MCF-7 breast cancer cells. J Biol Chem 286: 18633–18640. 21464128 10.1074/jbc.M111.220756 PMC3099679

[48] Verdonk ML, Cole JC, Hartshorn MJ, Murray CW, Taylor RD (2003) Improved protein-ligand docking using GOLD. Proteins: Structure, Function, and Genetics 52: 609–623. 10.1002/prot.10465 12910460

[49] Kellogg GE, Abraham DJ (2000) Hydrophobicity: is LogP(o/w) more than the sum of its parts? Eur J Med Chem 35: 651–661. 10960181 10.1016/s0223-5234(00)00167-7

[50] Marabotti A, Spyrakis F, Facchiano A, Cozzini P, Alberti S, et al (2008) Energy-based prediction of amino acid-nucleotide base recognition. J Comput Chem 29: 1955–1969. 18366021 10.1002/jcc.20954

[51] Hamada Y, Yamada I., Uenaka M, Sakata T (1993) Method for preparing benzoic acid derivatives. Patent US5214202.

[52] Lee BD, French KJ, Zhuang Y, Smith CD (2003) Development of a syngeneic in vivo tumor model and its use in evaluating a novel P-glycoprotein modulator, PGP-4008. Oncol Res 14: 49–60. 14552591 10.3727/000000003108748603

[53] Larkin MA, Blackshields G, Brown NP, Chenna R, McGettigan PA, et al (2007) Clustal W and Clustal X version 2.0. Bioinformatics 23: 2947–2948. 17846036 10.1093/bioinformatics/btm404

[54] Maceyka M, Harikumar KB, Milstien S, Spiegel S (2012) Sphingosine-1-phosphate signaling and its role in disease. Trends Cell Biol 22: 50–60. 22001186 10.1016/j.tcb.2011.09.003 PMC3253987

[55] Melo F, Sánchez R (2002) Sali (2002) A Statistical potentials for fold assessment. Protein Sci 11: 430–448. 11790853 10.1002/pro.110430 PMC2373452

[56] Puneet P, Yap CT, Wong L, Lam Y, Koh DR, et al (2010) SphK1 regulates proinflammatory responses associated with endotoxin and polymicrobial sepsis. Science 328: 1290–1294. 20522778 10.1126/science.1188635

[57] Shaner RL, Allegood JC, Park H, Wang E, Kelly S, et al (2009) Quantitative analysis of sphingolipids for lipidomics using triple quadrupole and quadrupole linear ion trap mass spectrometers. J Lipid Res 50: 1692–1707. 19036716 10.1194/jlr.D800051-JLR200 PMC2724058

